# Measuring dementia-related stigma in the Dutch general public: Translation and validation of the dementia public stigma scale

**DOI:** 10.1177/13872877261419067

**Published:** 2026-02-12

**Authors:** Anne A. C. Kolmans, Sascha R. Bolt, Ruslan Leontjevas, Wijnand A. IJsselsteijn, Debby L. Gerritsen

**Affiliations:** 1 Department of Primary and Community Care, 6034 Radboud University Medical Center, Radboud Institute for Medical Innovation, Radboudumc Alzheimer Center, Nijmegen, Netherlands; 2 Tranzo, Tilburg School of Social and Behavioral Sciences, 7899 Tilburg University, Tilburg, Netherlands; 3 Faculty of Psychology, 10198 Open University of the Netherlands, Heerlen, Netherlands; 4 Human-Technology Interaction, 3169 Eindhoven University of Technology, Eindhoven, Netherlands

**Keywords:** Alzheimer's disease, dementia, Netherlands, social stigma, translating, validation study

## Abstract

**Background:**

People with Alzheimer's disease or other types of dementia may experience stigma, which can influence their quality of life. Valid measurement instruments of public dementia-related stigma are lacking.

**Objective:**

We aimed to translate and validate the 16-item Dementia Public Stigma Scale (DePSS) in Dutch.

**Methods:**

A survey was conducted among a nationally representative sample of the Dutch population (n = 524). A subset (n = 145) completed the DePSS again after one month. Following validation guidelines, floor and ceiling effects, structural validity, internal consistency, and test-retest reliability were assessed. We used open-ended questions to investigate content validity. The responses provided insights into respondents’ perceptions of dementia and their interactions with people with dementia.

**Results:**

Forward-backward translation required minor adaptations. No floor or ceiling effects were observed. Confirmatory factor analysis indicated an acceptable fit (CFI = 0.988, RMSEA = 0.073, SRMR = 0.065). Internal consistency (α = 0.82, ω = 0.79) and test-retest reliability (ICC = 0.82, 95%CI 0.76–0.89) were good, with no significant differences between test and retest scores (t(144) = 0.135, p = .893). Responses to open-ended questions were largely clustered under DePSS items, indicating good content validity. Additional themes were disconnection from present reality; feeling pity for people with dementia; and manifestations of negative emotions.

**Conclusions:**

The Dutch DePSS demonstrated good psychometric properties. Together with other versions, these findings enhance the generalizability of the DePSS across diverse populations. Further validation and application of the DePSS will help deepen our understanding of dementia-related stigma and may also inform stigma reduction interventions.

## Introduction

There are more than 50 million people with dementia worldwide,^1^ approximately 300,000 of whom currently reside in the Netherlands.^2^ Public stigma can negatively impact quality of life for people with dementia.^3–5^ In response, awareness of dementia-related stigma in the Netherlands is growing,^2^ and several stigma reduction strategies and dementia-friendly initiatives are currently being deployed.^6,7^ Despite these efforts, dementia-related stigma remains a prominent issue.^8^ In a survey conducted in 2024, 11.4% of the Dutch general public indicated that they perceived people with dementia as dangerous and 67.6% believed that people with dementia are impulsive and unpredictable, which are both common stereotypes associated with dementia.^9^

The attribution theory describes public stigma as the endorsement of negative stereotypes and behaviors towards a stigmatized group by a large social group. The theory suggests that public stigma consists of three aspects: stereotypes (i.e., negative beliefs about stigmatized groups), prejudice (i.e., negative emotional reactions to stereotypes), and discrimination (i.e., behavioral consequences of prejudice).^10,11^ Hence, public stigma occurs when multiple people within a community have negative beliefs about people with dementia (stereotypes), harbor negative feelings towards people with dementia due to these stereotypes (prejudice), and subsequently treat people with dementia unfairly (discrimination). All three aspects of stigma have been found in relation to people with dementia. For example, studies have shown that people with dementia may be perceived as dangerous (stereotypes),^12^ that they may elicit fear^13^ or anxiety in others (prejudice),^12^ and that they may be socially avoided (discrimination).^14,15^ Consequently, public dementia-related stigma may lead to a decreased quality and quantity of social interactions and relationships of people with dementia.^16^

Evaluating public stigma and its impact on the lives of people with dementia requires valid assessment tools. However, no “gold standard” exists for measuring public dementia-related stigma.^8^ As a result, studies have adopted many different measures,^5,8^ hindering cross-study comparisons. Previous research has often used assessment tools that lack a theoretical background.^17^ Additionally, even though dementia-related stigma differs from stigma associated with other conditions (e.g., mental health problems or AIDS), many studies still use tools that were originally developed for other conditions.^8^ Instruments based upon a theoretical framework and developed specifically for assessing dementia-related stigma could facilitate a better understanding of the factors underpinning stigma and the factors that may help reduce it.^17^

The Dementia Attitudes Scale (DAS) measures dementia-related stigma using two factors, namely, social comfort (prejudice and discrimination) and dementia knowledge (stereotypes).^18^ The DAS has already been translated and validated into Dutch^19^ and Croatian.^20^ The original version of the DAS was validated using a sample of medical students. In contrast, the Dutch and Croatian versions of the DAS were validated using a sample of the general public and showed large differences in terms of factor loadings compared to the original.^19,20^ This suggests that the DAS is not fit to measure dementia-related stigma in the general public. Hence, other measurement tools should be considered for measuring public stigma associated with dementia in the Dutch context.

The Dementia Public Stigma Scale (DePSS) is a possible alternative instrument. Based on the attribution theory,^21^ the DePSS focusses on stigma related to dementia in general rather than stigma related to particular types of dementia (such as Alzheimer's disease). An initial psychometric evaluation of the DePSS was conducted as part of its development.^21^ However, although the instrument showed promising psychometric properties, this evaluation had some limitations. First, since a convenience sample was used, respondents likely had an interest in dementia, and the sample consisted mostly of middle-aged women with relatively high levels of education and individuals who knew someone with dementia. Second, test-retest reliability and concurrent validity were not examined. The DePSS was first published in December 2021 and has already been translated into Chinese,^22^ Italian,^23^ and Korean.^24^ The Italian and Korean versions showed good psychometric properties.^23,24^ A Dutch version does not yet exist.

Stigma is a complex construct which is shaped by socio-cultural contexts.^25^ It is important to translate and adapt dementia-related stigma scales, such as the DePSS, into diverse languages.^22^ Considering that the awareness of dementia-related stigma in the Netherlands is growing,^2^ and the DePSS was specifically developed to measure dementia-related public stigma, it could be a useful tool to evaluate stigma reduction strategies.^21^ Therefore, the aim of this study was to translate the DePSS into Dutch and to assess the psychometric properties of the translated instrument.

## Methods

### Study design

This study was part of a larger survey study that aimed to investigate dementia-related public stigma and associated factors in the Netherlands. We used an online survey to validate the Dutch version of the DePSS, applying international validation guidelines. Specifically, we followed the COnsensus-based Standards for the selection of health Measurement INstruments (COSMIN)^26,27^ and the primer for best practices for developing and validating scales.^28^ These criteria were developed to assess measurement properties of scales, such as structural validity, reliability, and internal consistency.^26,28^

### Setting

Respondents for the online survey were recruited via the Longitudinal Internet studies for the Social Sciences (LISS panel), which is managed by the non-profit research institute Centerdata (Tilburg University, the Netherlands).^29^ The LISS panel is a representative sample of Dutch individuals who participate in monthly internet surveys on varying topics. The panel is based on a true probability sample of households drawn from the population register by Statistics Netherlands.^30^

### Participants

For our sample, we followed the convention of recruiting at least 10 persons per item.^28^ All LISS-panel members were eligible for this study, with the exception of individuals with dementia, as the DePSS items were considered potentially confronting for people with dementia. This exclusion criterion was assessed at the start of the survey by asking respondents about their familiarity with dementia, including whether they had dementia themselves. For the survey, which took place in December 2023, a random selection of 670 respondents was used. For the retest, 160 of those respondents were randomly selected to complete the DePSS a second time in January 2024.

Centerdata abides by the European General Data Protection Regulation (GDPR).^29^ This study was conducted in agreement with the Declaration of Helsinki and the applicable Dutch laws and codes of conduct. The survey received approval from the ethics committee of the LISS panel (for details, see https://www.lissdata.nl/ethics). The Medical Ethics Review Committee (CMO Arnhem-Nijmegen region) declared that the survey study is not subject to the Medical Research Involving Human Subjects Act (WMO) (File number 2023–16741). Participation was voluntary and anonymous.

### Measurement

#### The instrument and translation into Dutch

The DePSS was developed by drawing items from existing tools (i.e., DAS),^18^ and creating additional items informed by existing literature.^21^ The items were reviewed for content validity and clarity of expression by the investigating team. The initial scale consisted of 47 items. Exploratory factor analysis resulted in a 16-item scale, loading on five factors. Cronbach's alpha values for the five factors and overall scale were moderate to high, suggesting that the five factors are different aspects of the same underlying construct (dementia-related public stigma).^21^ Tests of measurement invariance indicated that the items operated equivalently across gender and exposure groups (i.e., being acquainted with someone with dementia or not).

The 16-item DePSS measures public dementia-related stigma in terms of stereotypes, prejudice, and discrimination.^21^ The scale consists of five factors. Prejudice is measured with one factor: fear and discomfort (4 items). Stereotypes are measured with three factors: incapability and loss (5 items), acknowledgment of personhood (3 items), and burden (2 items). Discrimination is measured with one factor: exclusion (2 items). Respondents are asked to indicate to what extent they agree with each item on a 7-point Likert scale. Response options range from 1 = *strongly disagree* to 7 = *strongly agree*. The scale includes six reversed items, which are reverse coded prior to the analyses. The total DePSS score is the sum of the 16 items (range 16–112), with higher scores indicating more stigma.

The quality of the development study of the DePSS was assessed using the COSMIN guideline (see Supplemental Material).^27^ The requirements were assessed by two researchers independently (AK and SB). Thereafter, the ratings were compared and discussed. Most requirements were rated as adequate or very good. However, the quality of the sample was assessed as doubtful, and the development study did not include qualitative methods for item development. Considering all requirements, the quality of the scale development was considered sufficient.

We translated the original English scale using elements of the forward-backward translation technique.^31^ The scale was translated into Dutch by a bilingual speaker (native Dutch and English), who aimed to maintain the essence of the original items while considering accuracy and nuances in Dutch. Then, the Dutch version was translated backwards into English by another bilingual speaker (native Dutch and fluent in English) to check against the source material. The back-translation was reviewed by the original scale's developer (SK) to ensure that the meaning of the items had not been lost. Subsequently, the research team compared and discussed both translations and the original scale. Final discrepancies were resolved with minor adjustments to the Dutch wording for four of the items.

#### Other measures

In addition to the DePSS, the survey included open-ended questions that assessed respondents’ own descriptions of people with dementia. We used answers to these questions to explore whether potential forms of dementia-related stigma were missing from the DePSS. The question “How would you describe a person with dementia in one or two sentences?” was used to explore other stereotypes. Additionally, respondents who were familiar with people with dementia either at work or in private life were asked: “How do you interact with people with dementia?” This question was used to explore forms of discrimination. Demographic information, such as respondents’ age and level of education, was available from the LISS panel.

### Data analysis

Floor and ceiling effects were examined for respondents who filled in all 16 items of the DePSS. If more than 15% of these respondents have the lowest or highest possible total score, this could indicate that items are missing at the extreme ends of the scale, which limits its content validity.^32^ Additionally, respondents’ answers to the open-ended questions were analysed using qualitative content analysis. First, deductive coding was used to classify data directly corresponding to the DePSS items. Second, inductive coding was used to identify additional potentially stigmatic themes. The first author (AK) performed deductive coding, and the coded data was cross-checked by the second author (SB). Discrepancies were discussed and resolved during reflective meetings. For inductive analysis of the remaining data, both authors performed open coding and subsequently discussed their coding to reach consensus and cluster codes into themes. Responses which stated that the respondent's image varied due to the large heterogeneity between people with dementia were not considered. Moreover, responses that specifically described a respondent's own lived experience with a person with dementia were also not considered, because stigma is partly caused by the generalization of the image sometimes seen in later stages of dementia. Respondents’ descriptions were coded when they were in line with the 16 items of the DePSS, or if they contradicted them. For example, when respondents described people with dementia as “still the same person as before the disease” this was also coded as pertaining to incapability and loss (corresponding to the item: “*People with dementia are no longer themselves because they have dementia”).*

Structural validity of the DePSS was assessed using Confirmatory Factor Analysis (CFA),^26,28^ within which the comparative fit index (CFI) is considered sufficient above 0.95, the root mean square error of approximation (RMSEA) is considered sufficient below 0.06, and the standardized root mean square residual (SRMR) is considered sufficient with values below 0.08.^26,28,33^

To assess the scale's internal consistency, we examined whether the items measured one underlying construct using Cronbach's Alpha and McDonalds Omega. Both were considered sufficient with values above 0.70.^26,28^

To assess test-retest reliability, we used a subset of respondents who completed the survey twice: once at baseline and once after one month. The intraclass correlation coefficient (ICC(3,1)) was calculated, which was considered sufficient with values above 0.70.^26^ To assess agreement between baseline and retest, one sample t-tests were used for both the total scale score and the score for each item.

Statistical data analyses were performed using IBM SPSS statistics version 29.0,^34^ and CFA was performed using JASP 0.18.3.^35^

## Results

### Translation from English into Dutch

Some adaptations were made to the DePSS to ensure that the scale would be accurate in the Dutch context. We changed “personal choices” (“*persoonlijke keuzes”*) into “their own choices” (“*hun eigen keuzes”*) to improve clarity and coherence in Dutch. Furthermore, we changed “I would…” (“*ik* zou”) into “I tend to…” (“*ik ben geneigd om”*) because this formulation aligned more with the other items.

### Participant characteristics

Out of the 670 panel members approached, 553 (82.5%) completed the baseline questionnaire. For the retest, 160 respondents who had completed the baseline measurement were randomly selected. Of them, 96.3% (154) completed the second questionnaire. Two respondents were excluded from the DePSS, since they had dementia. A total of 27 respondents (9 for the retest) were excluded from the data analysis based on conflicting answers on the survey (e.g., respondents who indicated that they did not know anyone with dementia, while answering another question referring to knowing someone with dementia). This inconsistency raised concerns about the reliability of their data, leading to their exclusion from further analysis. As a result, data from 524 respondents (145 for the retest) were included in the analysis.

Out of 524 respondents, 277 were female and 247 were male, with ages ranging from 16 to 93 (*M* = 54.2, *SD* = 17.6). Level of education reported by respondents were as follows: lower education 24.6% (i.e., elementary school, or secondary school preparing for vocational education); intermediate education 34.7% (i.e., secondary school preparing for (applied) university, or vocational education); and higher education 40.7% (i.e., (applied) university degree). For the retest, 73 of 145 respondents were female and 72 were male, with ages ranging from 17 to 84 (*M* = 53.1, *SD* = 17.5). Respondents’ reported levels of education were lower 24.8%, intermediate 35.8%, and higher 39.3%.

### Content validity

There were no floor and ceiling effects, as none of the participants had the lowest (16) nor the highest possible score (112). Respondents’ answers to open-ended questions could largely be clustered under the DePSS items (see Table 1). Their answers frequently involved notions of incapability and loss, and acknowledgment of personhood—both stereotypes. The other DePSS factors were less commonly mentioned. Respondents’ answers to the open-ended questions both aligned with and contradicted three of the five DePSS factors (i.e., incapability and loss, acknowledgement of personhood, and exclusion). For instance, some respondents described people with dementia as childlike, whereas other respondents emphasized that people with dementia are adults who should be treated accordingly. Furthermore, different portrayals associated with possible stigma not included in the DePSS were identified. A common portrayal was the disconnection from present reality: respondents described people with dementia as living in their own world, reverting to the past, or having a vacant stare. Another recurrent theme was feeling pity for people with dementia. People with dementia were described as being lonely and respondents indicated that they felt pity for them. The last theme was manifestations of negative emotions, where people with dementia were described as showing anxiety, anger, and sadness.

**Table 1.** Example quotes and frequency of the DePSS factors and additional themes in answers to the open-ended questions.

	Example quote DePSS Factors	Spontaneously reported, N
Fear and discomfort	“It [interacting with people with dementia] makes me insecure and gives me an unpleasant feeling.”	6
Incapability and loss	“Someone who is slowly losing grip on his or her life and who is becoming increasingly dependent on others.”	139*
Acknowledgment of personhood	“[When interacting with people with dementia] I try to make them as happy as possible, to ensure that they can still enjoy life to the fullest.”	198*
Burden	“Someone who, unfortunately, can no longer be themself and has a disease that affects those around them more than the person with dementia.”	5
Exclusion	“I will continue to accept direct family members [with dementia], but I try to avoid strangers.”	4*
Additional Themes		
Disconnection from present reality	“Someone in the present, who lives in his past.”	162
Feeling pity for people with dementia	“An utterly helpless person who endures nearly the worst and most inhumane thing that exists.”	27
Manifestations of negative emotions	“That [a person with dementia] is a person who is helpless and scared because they don’t understand anything anymore and therefore they get angry.”	47

*Frequencies include both positive and negative notions of dementia.

### Structural validity

The CFA indicated an acceptable fit to the data: χ^2^ was 358.61 (df = 94, *p* < 0.001), CFI = 0.988, RMSEA = 0.073 (95%CI [0.065, 0.081]), and SRMR = 0.065. Although RMSEA was larger than 0.06, it was below 0.08 indicating an acceptable fit to the data. Factor loadings ranged from 0.550 to 0.952 (see Table 2). All factor loadings were statistically significant (*p* < 0.001), indicating a strong association between the observed items and their original factors.

**Table 2.** CFA results for the Dutch DePSS on the original five-factor model.

	Original DePSS items	Item code	Factor loading
Fear and Discomfort	I feel confident around people with dementia (R)	FD1	0.827
	I am comfortable touching people with dementia (R)	FD2	0.907
	I feel relaxed around people with dementia (R)	FD3	0.907
	I am afraid of people with dementia	FD4	0.625
Incapability and Loss	People with dementia should always be supervised	IL5	0.550
	People with dementia are unpredictable	IL6	0.711
	People with dementia are very much like children	IL7	0.624
	People with dementia are incapable of making any personal decisions	IL8	0.747
	People with dementia are no longer themselves because they have dementia	IL9	0.667
Acknowledgment of Personhood	People with dementia can enjoy life (R)	AP10	0.734
	People with dementia can feel when others are kind to them (R)	AP11	0.758
	It is possible to enjoy interacting with people with dementia (R)	AP12	0.786
Burden	People with dementia are a burden to their family	B13	0.866
	People with dementia are a burden on the healthcare system	B14	0.759
Exclusion	I would exclude people with dementia from activities	E15	0.867
	I would ignore people with dementia	E16	0.952

### Internal consistency

At baseline and retest, Cronbach's Alpha showed high internal consistency for the total DePSS score (0.819 and 0.859 respectively). Furthermore, at baseline and retest McDonalds Omega also showed high internal consistency (0.793 and 0.840 respectively). Cronbach's Alpha and McDonalds Omega for the five factors at baseline and retest were all sufficient: range 0.740–0.918 (see Table 3).

**Table 3.** Internal consistency (Cronbach's Alpha and McDonalds Omega) per factor for the baseline and retest.

Factor	Baseline		Retest	
	α	ω	α	ω
Fear and Discomfort	0.848	0.875	0.897	0.918
Incapability and Loss	0.750	0.746	0.780	0.779
Acknowledgment of personhood	0.740	0.741	0.749	0.753
Burden	0.752		0.780	
Exclusion	0.864		0.873	

### Test-retest reliability

The ICC(3,1) indicated good agreement between test and retest scores: 0.82 for single measures (95%CI [0.759, 0.867]). For the total score, the paired sample t-test showed no significant differences between test (*M* = 54.80, *SD* = 11.17) and retest (*M* = 54.72, *SD* = 11.39), *t*(144) = 0.135, *p* = 0.893. Table 4 shows the results of the paired sample t-tests per item. For only 2 out of 16 items did the t-tests indicate significant differences: “I feel confident around people with dementia” (*t*(144) = 2.098, *p* = 0.038) and “I would exclude people with dementia from activities” (*t*(144) = −2.571, *p* = 0.011). Taken together, the ICC and paired sample t-tests suggest good test-retest reliability.

**Table 4.** Results of paired samples T-tests examining the test-retest reliability per item (N = 145).

	Baseline		Retest				95% CI	
Item	Mean	SD	Mean	SD	t	p	Lower	Upper
FD1	3.39	1.459	3.17	1.443	2.098	0.038	0.013	0.429
FD2	3.35	1.530	3.17	1.458	1.866	0.064	−0.011	0.383
FD3	3.34	1.512	3.23	1.423	1.024	0.307	0.108	−0.103
FD4	2.28	1.240	2.29	1.154	−0.071	0.943	−0.198	0.184
IL5	4.84	1.380	4.86	1.318	−1.057	0.292	−0.337	0.102
IL6	4.80	1.078	4.72	1.070	0.799	0.426	−0.112	0.263
IL7	4.01	1.346	4.14	1.310	−1.264	0.208	0.104	−0.336
IL8	4.52	1.329	4.46	1.318	0.668	0.505	−0.122	0.246
IL9	4.77	1.262	4.76	1.215	0.134	0.894	−0.190	0.218
AP10	2.55	1.013	2.59	1.121	−0.458	0.628	−0.210	0.127
AP11	2.48	1.074	2.34	0.960	1.621	0.107	−0.029	0.291
AP12	2.70	1.271	2.66	1.215	0.315	0.754	−0.182	0.251
B13	3.71	1.404	3.72	1.250	−0.138	0.890	−0.211	0.183
B14	3.49	1.528	3.60	1.421	−1.079	0.282	−0.312	0.092
E15	2.46	1.143	2.68	1.252	−2.571	0.011	−0.378	−0.049
E16	2.22	1.164	2.34	1.070	−1.814	0.072	−0.259	0.011

## Discussion

This study aimed to translate the DePSS^21^ into Dutch and to validate the resulting Dutch version of the instrument. The Dutch version of the DePSS demonstrated good psychometric properties. The CFA supported the original 5-factor model in a large representative sample of the Dutch population. Internal consistency for the overall scale and its factors was high and test-retest reliability was strong. Content validity was acceptable as supported by the quality of the scale development process according to the COSMIN guidelines^26^ and the qualitative analysis of the open-ended questions. Furthermore, floor and ceiling effects were absent.

The development of the original DePSS items was informed by existing tools and literature,^21^ without involvement of qualitative methods. To overcome this, and to investigate content validity, we used open-ended questions to assess respondents’ own descriptions of people with dementia and their interactions with them. Respondents’ descriptions largely aligned with the DePSS content, suggesting that the DePSS covers a range of stigmatizing assumptions that the general public may have. Additionally, respondents’ descriptions of people with dementia both aligned with and contradicted the stigmatic statements of the DePSS, showing the complexity of dementia-related stigma. However, qualitative analysis of the open-ended questions showed the following additional themes that may reflect dementia-related stigma: disconnection from present reality, feeling pity for people with dementia, and manifestations of negative emotions.

The themes disconnection from present reality and manifestations of negative emotions correspond to stereotypical portrayals of people with dementia. Disconnection from present reality and an inability to adequately communicate are commonly depicted in literature and media about people with dementia.^36–38^ Additionally, pity towards people with dementia, which was sometimes reported by respondents, is a sentiment often classified as prejudice^10,11^; this has also been reported in other studies.^13,39^ While many stereotypical portrayals of people with dementia were described, respondents mentioned forms of prejudice or discrimination less often. For example, only a few respondents mentioned experiencing feelings of fear and discomfort when interacting with people with dementia, which is measured in the DePSS.

The lower levels of reported prejudice and discrimination, as compared to stereotypes, may be partially explained by social desirability bias, which is a common issue in stigma research^18,40^ and self-reported measures.^41,42^ Although behaviors may be most observable from the outside, people may not accurately describe their own behaviors when using a self-report survey, especially when these behaviors can be considered dubious or inconsistent with societal norms (e.g., discriminatory behaviors towards people with dementia).^42^ An additional explanation comes from the nature of the different aspects of stigma.^10,11^ In the context of dementia, stigma occurs when an individual internalizes negative beliefs about a person with dementia (stereotypes), harbors negative feelings towards the person due to these stereotypes (prejudice), and subsequently treats the person unfairly (discrimination). Hence, stereotypes encompass the outermost level of stigma, while prejudice depends on the internalization of these stereotypes, and discrimination on the presence of both stereotypes and prejudice. Therefore, in line with the results of our qualitative analysis, people may be most likely to hold and report stereotypes. Furthermore, as social desirability is a common issue in stigma research, we recommend including a social desirability scale in future applications of the DePSS.

Similar to our findings from the qualitative analyses, the DePSS primarily measures stereotypes of people with dementia. Specifically, 3 out of 5 factors measure stereotypes involving: incapability and loss (5 items), acknowledgment of personhood (3 items), and burden (2 items).^21^ In contrast, only one factor measures prejudice (fear and discomfort: 4 items) and one factor measures discrimination (exclusion: 2 items). Although the DePSS effectively covers critical content of dementia-related stigma, the qualitative analyses points to stigmatic notions that could be further explored. However, adding items to the DePSS should be considered carefully, as it is important to balance the assessment burden with the need to capture all relevant information.^8^ Given that dementia-related stigma is a complex, manifold construct, it is difficult to measure all forms of stigma, keeping in mind the practicality of the scale. Considering the DePSS in its current form measures a range of important aspects of dementia-related stigma, while remaining relatively short, we do not suggest adding any additional items.

Besides content validity, this study demonstrated good internal consistency, test-retest reliability, and structural validity. The internal consistency of the overall scale at baseline and retest (Cronbach's α 0.819 and 0.859, respectively) was comparable to the development study (Cronbach's α = 0.818)^21^ and the Dutch DAS (Cronbach's α = 0.858).^19^ Additionally, while factor analysis of the Dutch DAS revealed differences from the original,^19^ the Dutch DePSS showed a factor structure consistent with the original scale, supporting its structural validity. Interestingly, the factor analysis of the Korean DePSS indicated a four-factor structure, which did not include the factor “exclusion”.^24^ This omission may reflect a social desirability bias, as well as a cultural inclination to refrain from expressing negativity. Furthermore, the Italian version of the DePSS also showed promising results for clarity, cultural appropriateness, and relevance.^23^

Hence, the DePSS, in its current form, can be viewed as a representative and valuable scale that measures relevant aspects of public stigma towards people with dementia. Given the primary focus on stereotypes of people with dementia, the DePSS could be a useful tool to assess interventions specifically targeted at reducing stereotypes or improving the imaging of people with dementia.

### Strengths and limitations

Two limitations of the original validation of the DePSS (i.e., the use of a convenience sample and the absence of test-retest reliability assessment) were addressed in this study.^21^ Our study used a large, high-quality, representative sample of the Dutch population and demonstrated strong test-retest reliability. This supports the stability of the DePSS over time, which is crucial for evaluating the effectiveness of stigma-reduction interventions.

Although this study shows robust support for the Dutch DePSS through its multi-method validation and use of a representative sample, some limitations must be mentioned. Criterion and convergent validity were not assessed. Currently, there is no ‘gold standard’ for assessing dementia-related public stigma,^5,8^ which complicates the execution of such validations. The Korean DePSS, however, demonstrated convergent validity through significant associations with fear of dementia, dementia attitudes, ageism, and anxiety about aging.^24^ Construct validity could be examined in future studies by comparing the DePSS with instruments that measure related constructs. Additionally, the assessment of content validity through respondents’ own descriptions of people with dementia was limited by their emphasis on stereotypes, while there were only a few notions of prejudice or discrimination. This limited the assessment of other forms of prejudice or discrimination absent in the DePSS. Even though the second open-ended question asked specifically about behaviour towards people with dementia, respondents still predominantly mentioned stereotypes. This raises the question of whether a survey is fit to investigate spontaneous descriptions of prejudice or discrimination or whether other methods would be more applicable (e.g., observations or in depth interviews).^40^ Moreover, cultural specificity should be considered. Validation took place within the Dutch context, so there is no certainty that the results are transferable outside the Netherlands. While the Chinese translation did not involve validation,^22^ the Italian,^23^ and Korean DePSS^24^ showed promising results. Furthermore, as evidence for the psychometric properties of the Dutch DePSS currently derives solely from this study, it is important to acknowledge that additional research is necessary to further validate these findings. However, together with the validation of the Korean and Italian DePSS, the promising results of the Dutch version of the DePSS support the scale's generalizability across different languages and cultural contexts. Nevertheless, additional research is needed to confirm the generalizability of the DePSS. This is especially relevant given the cultural variations in stigma.^25^ The use of cross-culturally comparable instruments are essential to measure stigma across different cultural and national contexts. Future studies could take these considerations into account when validating the DePSS in other languages and contexts.

### Conclusion

In conclusion, this study indicates that the Dutch version of the DePSS has good psychometric properties. The study fills a critical gap, since valid instruments to measure public dementia-related stigma were lacking in the Netherlands. The Dutch DePSS is a reliable and valid instrument that can be employed in research and practice to assess and address dementia-related public stigma in the Netherlands. It could serve to evaluate the effects of stigma reduction interventions aimed at improving public attitudes towards people with dementia. However, as evidence for the psychometric quality of the Dutch translation of the DePSS is derived from this single study, further research is required to confirm its reliability and enhance its practical applicability. In addition, further validation and application of the DePSS in diverse contexts and languages may deepen our understanding of public dementia-related stigma and inform stigma reduction interventions around the world.

## Supplemental Material

sj-docx-1-alz-10.1177_13872877261419067 - Supplemental material for Measuring dementia-related stigma in the Dutch general public: Translation and validation of the dementia public stigma scale Supplemental material, sj-docx-1-alz-10.1177_13872877261419067 for Measuring dementia-related stigma in the Dutch general public: Translation and validation of the dementia public stigma scale by Anne A. C. Kolmans, Sascha R. Bolt, Ruslan Leontjevas, Wijnand A. IJsselsteijn and Debby L. Gerritsen in Journal of Alzheimer's Disease
