# Supplementary material for: Measuring dementia-related stigma in the Dutch general public: Translation and validation of the dementia public stigma scale
Source: J Alzheimers Dis. 2026 Feb 12;110(2):956–65. doi: 10.1177/13872877261419067 (PMC12982547; doi:10.1177/13872877261419067)
Supplement: sj-docx-1-alz-10.1177_13872877261419067 - Supplemental material for Measuring dementia-related stigma in the Dutch general public: Translation and validation of the dementia public stigma scale [file sj-docx-1-alz-10.1177_13872877261419067.docx]

**Supplemental Material**

**Measuring dementia-related stigma in the Dutch general public: Translation and validation of the Dementia Public Stigma Scale**

**Evaluation of the quality of the development study**

The COSMIN guideline provides standards for evaluating measurement properties of outcome measurement instruments,^1^ such as for evaluating the quality of the development study of a measure. Although the DePSS is not a patient-reported outcome measure (for which this guideline is specified), this guideline can be used to assess the quality of the scale development. The standards for evaluating the quality of the development consists of two parts: 1a) Prom Design and 1b) Cognitive interview study or other pilot test. Since no cognitive interview or study pilot test was performed, only part 1a (Prom design) was assessed in this study (see Supplemental Table 1). The requirements were assessed by two researchers independently (SB and AK). Each requirement was rated as V= very good, A = adequate, D = doubtful, I = inadequate, or N = not applicable. Thereafter, the ratings were compared and discussed until a consensus was reached.

All five general design requirements were rated the same by the two researchers. The quality of the sample of the scale development study was, however, assessed as doubtful. Although the study had a large sample from diverse backgrounds, respondents were likely individuals with an interest in dementia. They used a convenience sample, recruiting respondents who were participating in a dementia educational program online, and their sample consisted mostly of middle-aged women with relatively high educational levels. Since this subgroup tends to display less dementia-related stigma than do men or those in older age groups,^2-4^ it is doubtful that the sample is representative for the general public.

For the concept elicitation, seven out of eight requirements were rated the same by the two researchers. For the other requirement consensus was reached. Most of the requirements were not applicable for the development study. The sample size of the development study was deemed appropriate. However, it was doubtful that an appropriate qualitative data collection method was used to identify relevant items for the new scale. Items were developed by examination of existing tools and created by the first author in line with existing literature.^5^ Given that no qualitative methods were used in the development of the items, this was rated as doubtful. Considering the COSMIN guidelines, the quality of the DePSS development would therefore be considered doubtful. However, given that the other requirements were all either adequate or very good, the quality of the development was considered sufficient.

**Supplemental Table 1.** Ratings of the General Design Requirements and Concept Elicitation of the DePSS Development of the Two Authors, and the Consensus.

| **1a. PROM design** | | | | |  |
| --- | --- | --- | --- | --- | --- |
| *General design requirements* | | **AK** | **SB** | **Consensus** | |
| 1 | Is a clear description provided of the construct to be measured? | V | V | V | |
| 2 | Is the origin of the construct clear: was a theory, conceptual framework or disease model used or clear rationale provided to define the construct to be measured? | V | V | V | |
| 3 | Is a clear description provided of the target population for which the PROM was developed? | V | V | V | |
| 4 | Is a clear description provided of the context of use (i.e., discriminative, evaluative purpose, and/or predictive) | A | A | A | |
| 5 | Was the PROM development study performed in a sample representing the target population for which the PROM was developed? | D | D | D | |
| *Concept elicitation (relevance and comprehensiveness)* | | **AK** | **SB** | **Consensus** | |
| 6 | Was an appropriate qualitative data collection method used to identify relevant items for a new PROM? | D | D | D | |
| 7 | Were skilled group moderators/ interviewers used? | N | N | N | |
| 8 | Were the group meetings or interviews based on an appropriate topic or interview guide? | N | N | N | |
| 9 | Were the group meetings or interviews recorded and transcribed verbatim? | N | N | N | |
| 10 | Was an appropriate approach used to analyse the data? | A | N | N | |
| 11 | Was at least part of the data coded independently? | N | N | N | |
| 12 | Was data collection continued until saturation was reached? | N | N | N | |
| 13 | For quantitative studies: was the sample size appropriate? | V | V | V | |
|  | **SUBTOTAL QUALITY CONCEPT ELICITATION STUDY** *Lowest score of items 6-13* |  |  | **D** | |
|  | **TOTAL QUALITY OF THE PROM DESIGN** *Lowest score of items 1-13* |  |  | **D** | |

**References**

1. Terwee CB, Prinsen C, Chiarotto A, et al. *COSMIN methodology for assessing the content validity of PROMs – user manual*. 2018.

2. Blay SL and Toledo Pisa Peluso E. Public stigma: the community's tolerance of Alzheimer disease. *Am J Geriatr Psychiatry* 2010; 18: 163-171.

3. Kim S, Anstey KJ and Mortby ME. Who displays dementia-related stigma and what does the general public know about dementia? Findings from a nationally representative survey. *Aging Ment Health* 2023; 27: 1111-1119.

4. Cheng ST, Lam LCW, Chan LCK, et al. The effects of exposure to scenarios about dementia on stigma and attitudes toward dementia care in a Chinese community. *Int Psychogeriatr* 2011; 23: 1433-1441.

5. Kim S, Eccleston C, Klekociuk S, et al. Development and psychometric evaluation of the Dementia Public Stigma Scale. *Int J Geriatr Psychiatry* 2022; 37: 10.1002/gps.5672.
